# Rapid Cycle Deliberate Practice Training for Simulated Cardiopulmonary Resuscitation in Resident Education

**DOI:** 10.5811/westjem.17923

**Published:** 2024-02-09

**Authors:** Jaron D. Raper, Charles A. Khoury, Anderson Marshall, Robert Smola, Zachary Pacheco, Jason Morris, Guihua Zhai, Stephanie Berger, Ryan Kraemer, Andrew D. Bloom

**Affiliations:** * University of Alabama at Birmingham, Department of Emergency Medicine, Birmingham, Alabama; † University of Alabama at Birmingham, Department of Internal Medicine, Birmingham, Alabama; ‡ University of Alabama at Birmingham, Center for Clinical and Translational Science, Birmingham, Alabama; § University of Alabama at Birmingham, Department of Pediatrics, Birmingham, Alabama

## Abstract

**Background:**

Simulation-based medical education has been used in medical training for decades. Rapid cycle deliberate practice (RCDP) is a novel simulation strategy that uses iterative practice and feedback to achieve skill mastery. To date, there has been minimal evaluation of RCDP vs standard immersive simulation (IS) for the teaching of cardiopulmonary resuscitation to graduate medical education (GME) learners. Our primary objective was to compare the time to performance of Advanced Cardiac Life Support (ACLS) actions between trainees who completed RCDP vs IS.

**Methods:**

This study was a prospective, randomized, controlled curriculum evaluation. A total of 55 postgraduate year-1 internal medicine and emergency medicine residents participated in the study. Residents were randomized to instruction by RCDP (28) or IS (27). Stress and ability were self-assessed before and after training using an anonymous survey that incorporated five-point Likert-type questions. We measured and compared times to initiate critical ACLS actions between the two groups during a subsequent IS.

**Results:**

Prior learner experience between RCDP and IS groups was similar. Times to completion of the first pulse check, chest compression initiation, backboard placement, pad placement, initial rhythm analysis, first defibrillation, epinephrine administration, and antiarrhythmic administration were similar between RCDP and IS groups. However, RCDP groups took less time to complete the pulse check between compression cycles (6.2 vs 14.2 seconds, *P* = 0.01). Following training, learners in the RCDP and IS groups scored their ability to lead and their levels of anticipated stress similarly (3.43 vs 3.30, (*P* = 0.77), 2.43 vs. 2.41, *P* = 0.98, respectively). However, RCDP groups rated their ability to participate in resuscitation more highly (4.50 vs 3.96, *P* = 0.01). The RCDP groups also reported their realized stress of participating in the event as lower than that of the IS groups (2.36 vs 2.85, *P* = 0.01).

**Conclusion:**

Rapid cycle deliberate practice learners demonstrated a shorter pulse check duration, reported lower stress levels associated with their experience, and rated their ability to participate in ACLS care more highly than their IS-trained peers. Our results support further investigation of RCDP in other simulation settings.

Population Health Research Capsule What do we already know about this issue?

*Rapid cycle deliberate practice (RCDP) is a simulation strategy that uses iterative practice and coaching to achieve skill mastery and is effective in procedural instruction.*

What was the research question?

*Is RCDP or immersive simulation (IS) more effective in training residents to perform cardiopulmonary resuscitation (CPR)?*

What was the major finding of the study?

*RCDP shortens pulse checks, and learners reported less stress and greater confidence performing CPR.*

How does this improve population health?

*Resuscitation instruction based in RCDP shows promise as a tool to enhance residents’ mastery of lifesaving CPR skills.*

## INTRODUCTION

Despite advances in resuscitation science and training, cardiac arrest remains the third leading cause of death in the United States.^1^ Millions of clinicians receive Basic Life Support (BLS) and Advanced Cardiac Life Support (ACLS) training, yet patients’ survival rates vary considerably.^2^ ^,^ ^3^ ^,^ ^4^ Immediate recognition of cardiac arrest, high quality cardiopulmonary resuscitation (CPR), and timely defibrillation are the mainstays of care.^5^ ^,^ ^6^ Effective education is crucial to execute these principles, improve team performance, and enhance outcomes.^7^

Simulation-based medical education (SBME) is well established in medical training and graduate medical education (GME). In SBME, learners gain experience from a realistic clinical scenario without the possibility of causing harm to a patient.^8^ Learners are given the freedom to develop skills through practice and gain valuable feedback via debriefing. As a result, SBME has been associated with improved skill development and patient outcomes.^9^ ^,^ ^10^ ^,^ ^11^

Rapid cycle deliberate practice (RCDP) is an innovative simulation strategy that uses iterative practice and feedback to achieve skill mastery. Developed from Anders Ericsson’s work on deliberative practice, RCDP allows for advanced learning through repetition and skill refinement.^12^ It was originally described by Hunt in 2014 and implemented in pediatric resuscitation training.^13^ In RCDP, learners begin a simulated scenario, but in contrast to the classical post-simulation debrief, the case is frequently paused by the instructor. Each break serves as an opportunity for corrective instruction, coaching, feedback, and subsequent supervised repitition.^13^

Over the last decade there has been an increased focus on RCDP training in resuscitation, with most studies focused on pediatric trainees.^13^ ^,^ ^14^ When compared to the standard immersive simulation (IS) approach, RCDP has demonstrated shorter time to initial chest compression and defibrillation in pediatric medicine trainees, improved chest compression fraction in adult medical trainees, and better skill retention.^13^ ^,^ ^14^ ^,^ ^15^ ^,^ ^16^ Even more recently, we have seen RCDP implemented into procedural training where it has also demonstrated positive learner outcomes. Groups trained in RCDP demonstrated better preparedness for intubation and post-procedure care in pediatric airway management.^17^ Similarly, RCDP-based training has been suggested for the donning and doffing of personal protective equipment, and our obstetric colleagues have proven its utility for forceps-based deliveries.^18^ ^,^ ^19^

Instruction based in RCDP has strong evidence to support its use in areas of medical education that are algorithmic in nature, and/or require a high degree of procedural skill. The American Heart Association (AHA) recognized this as recently as 2020, recommending that deliberate practice be incorporated into BLS and ACLS training, simultaneously identifying it as an educational strategy warranting further research.^7^ Despite this call to action, there has been a paucity of literature evaluating RCDP in ACLS training for the care of adult patients, regardless of learner type.^20^ We sought to address this knowledge gap through the evaluation of RCDP for ACLS as it is applied to postgraduate year (PGY)-1 residents in GME. We did this through a comparison of time to completion of critical ACLS actions between RCDP and IS groups (our primary objective). As a secondary objective, we compared resident perceptions between RCDP- and IS-trained groups.

## METHODS

### Study Design

In July 2022, we conducted a prospective, randomized, controlled study approved by the institutional review board.

### Setting and Participants

The study was conducted in an accredited simulation center that is part of a large academic teaching hospital and involved 43 internal medicine (IM) and 12 emergency medicine (EM) PGY-1 residents who had obtained ACLS certification in the two weeks preceding this study. No other coaching or instruction regarding the care of a pulseless patient was provided prior to study implementation. All 55 residents participated voluntarily in the study. Faculty facilitators of all simulation sessions were IM and EM faculty who were board certified in their respective fields. Each facilitator underwent formal IS- and RCDP-facilitator training prior to involvement in the study. Facilitators were not blinded to the study objectives.

### Protocol

In the week prior, residents were provided with a description of the study and an electronic copy of the informed consent document to allow for a detailed and private review. Each of the 55 participants then provided written informed consent on the date of their scheduled simulation event. Our study used five teams for each instructional intervention. Each team was comprised of five or six members who were randomly assigned to either RCDP or IS, for a total of 55 participants (28 in RCDP groups, 27 in IS groups). While there was a fixed and limited number of available participants (IM and EM interns), we performed a post-hoc power analysis to establish a basis for future work. With an α = 0.05, this study had 29% power to detect a large effect size (d = 1) for primary outcomes and 71% power to detect a medium effect size (w = 0.3) for secondary outcomes (G*Power 3.1.9.7). We used an online randomization generator (https://www.randomizer.org/) to divide participants into 10 teams, with five teams for each instructional method.

Due to scheduling differences, IM and EM participants were separated and completed their respective experiences on different days. The IM faculty facilitated all IM resident sessions. To minimize confounding related to the effectiveness of the individual facilitator, the two IM faculty facilitators led both the RCDP and the IS sessions for the IM residents. The 12 EM participants completed their experience the following week in two teams of six, one of which was assigned to RCDP and the other to IS. The EM faculty facilitated both EM resident sessions. All faculty facilitators were trained in implementation of RCDP and IS. This training was provided by certified healthcare simulation instructors in our internationally accredited institutional simulation center. No faculty facilitators were involved in the extraction of performance data.

The same two embedded simulation participants (ESP) functioned as nurses for all sessions. The ESPs in all sessions were registered nurses and certified healthcare simulation educators employed by our institutional simulation center. The ESPs were instructed to assist only with care tasks when directly asked for specific task assistance (eg, locating care items) but did not trigger initiation of individual task completion or provide guidance on task performance.

Following informed consent, learners were asked to complete a pre-simulation survey to establish baseline learner characteristics. The survey queried each participant’s prior level of experience as well as self-perceived ability to lead and participate in the care of a pulseless patient. The surveys also assessed the learner’s anticipated and prior experienced stress associated with code leadership and participation. Each measure was assessed using a five-point Likert-type scale.

### Immersive Simulation Protocol

All IS teams were provided with the same scripted pre-brief, which described the basic tenets of simulation and informed participants that they would be caring for a pulseless patient. Teams were not instructed regarding the assignment of clinical roles but were allowed to self-assign as they deemed appropriate. The IS teams were then activated by an ESP functioning in the role of a nurse who brought the participants to the care area and asked participants to evaluate an unresponsive patient.

Once outside the patient’s room, participants assumed care for the patient without further coaching or intervention. The IS participants were permitted to navigate the patient’s case without interruption, while physician facilitators observed their actions from a simulation control room with audio and visual surveillance of the simulation area. The IS learners were allowed to navigate their case without interruption until the fourth pulse check or until 30 minutes had elapsed, at which time facilitators initiated return of spontaneous circulation and the case was terminated. Given the nature of the IS educational sessions, learners did not have the opportunity to rotate roles. Learners then returned to the briefing room, and physician facilitators debriefed based on observed performance according to a standardized debriefing guide and until total case time reached 45 minutes. The guide emphasized coaching regarding resuscitation and time-sensitive interventions that matched the primary outcome measures (eg, time to identification of pulselessness, time to initiation of chest compressions, etc).

### RCDP Simulation Protocol

All RCDP groups were given a standard pre-brief that described the basic tenets of simulation. Groups were then provided with an introduction to the simulation modality assigned to them. Teams were not instructed regarding the assignment of clinical roles but were allowed to self-assign as they deemed appropriate. The RCDP teams were activated by an ESP who brought the participants to the care area and asked them to evaluate an unresponsive patient while physician facilitators observed at the bedside. The RCDP groups rotated roles, allowing them the opportunity to direct the resuscitation and receive feedback.

In addition to their standardized training, all facilitators were provided with an RCDP coaching guide, which was focused on the same resuscitation and time-sensitive interventions as the immersive case debriefing guide. Facilitators provided real-time coaching and feedback based on the RCDP coaching guide. Cases were then restarted, rewound, or resumed according to facilitator discretion. Total learner simulation and debriefing time was 45 minutes for each RCDP case.

### Protocol Overlap

Upon completion of debriefing and closure of their respective cases, all participants returned to the briefing space. Maintaining separation of initial RCDP vs IS groups, a subsequent IS session was completed by all participants during which audio and visual recordings were obtained. Data abstraction of times to completion of critical ACLS actions was obtained from this session. Participants were activated a second time by the ESP to care for an additional, unresponsive patient. Learners were allowed to role assign and complete the case without intervention from the ESP or facilitator.

### Primary Outcome Measures

Time to completion of critical actions was used as a surrogate for proficiency in the performance of an ACLS-based resuscitation. These critical actions were defined by research team consensus after reviewing ACLS protocols. Time zero was determined based upon learner entry into the care area, and times to completion of resuscitative time-based interventions were extracted through video review by the primary investigator. To mitigate bias from faculty working with their own residents, data abstraction from video recordings was performed by the primary investigator, who was not involved in simulation session facilitation. The primary investigator was blinded to RCDP vs IS group assignment at the time of data abstraction. Times from room entry to first pulse check, first chest compression, backboard placement, defibrillator pad attachment, initial rhythm analysis, initial defibrillation, initial epinephrine administration, and antiarrhythmic administration were recorded. The duration of pause between compression cycles was also obtained for each session.

### Secondary Outcome Measures

Learners were queried using pre- and post-experience surveys, which were distributed in paper format immediately before and after the simulation sessions. We developed the surveys based on Kirkpatrick’s theory of educational training and evaluation, focusing primarily on level 1 and 2 analyses.^21^ All survey items used a 1–5 Likert-type scale to quantify all qualitative questions, and survey response rates for all surveys were 100%. Prior to the educational intervention, learners were asked to rate their self-perceived ability to participate in and ability to lead a code (1 not at all capable, to 5 extremely capable). They were also asked to rate their anticipated stress associated with participation and leadership of a code (1 not at all stressful, to 5 extremely stressful). Finally, they were queried regarding the number of simulated codes they had participated in or led, as well as the number of actual codes they had participated in or led.

Following the education intervention, learners were asked to again rate their self-perceived ability to participate in and lead a code. They were also asked to rate the overall effectiveness of their experience (1 not at all effective, 5 extremely effective). Finally, learners were asked to rate the stress level they perceived to be associated with participating and leading their simulated experience (1 not at all stressful, to 5 extremely stressful).

### Statistical Analysis

First, we compared prior simulated and genuine CPR experiences as leader and as participant for RCDP and IS groups, using the Cochran-Mantel-Haenszel test, given the ordinal nature of the Likert-type scale. We defined simulated experiences as those involving CPR training that did not involve the care of a patient. Genuine experiences were defined as those involving the CPR-based resuscitation of a coding patient. We then compared the time-based differences between RCDP and IS groups using a Student *t*-test or a Wilcoxon test when there was substantial deviation from normality. Our sample size for all primary outcome measures was 10 teams. We compared mean time differences between the two groups for first pulse check, first chest compression, pause duration, backboard placement, defibrillator pad placement, first rhythm analysis, first defibrillation, first epinephrine administration, and amiodarone administration.

Our sample size for all secondary outcome measures was 55 individuals. We also compared pre- and post-training survey data between the two groups using the Cochran-Mantel-Haenszel test given the ordinal nature of the Likert-type scale. The learner’s experience as code leader and participant and overall effectiveness of experience were also included in the post-training survey. Ability to lead, ability to participate, anticipated stress leading, and anticipated stress participating were included in both surveys. Finally, we compared stress leading and stress participating in pre- and post-training for both groups using a generalized Stuart-Maxwell test to evaluate the improvement after training.^22^ We used an alpha level of 0.05 for all statistical tests. A Benjamini-Hochberg false discovery rate adjustment was applied for multiple comparisons. All programs were written in SAS 9.4. (SAS Institute Inc, Cary, NC).

## RESULTS

### Prior Learner Experience

Prior learner experience was similar between the groups and did not appear to be a significant confounder (Table 1). The numbers of experiences are reported as medians with minimum and maximum values due to lack of normal distribution.

**Table 1.** Cardiopulmonary resuscitation experience prior to simulation.

	Group	Median	Min	Max	*P*-value
Simulation	IS	1	0	17	0.34
leader	RCDP	2	0	6	
Genuine	IS	0	0	40	0.81
leader	RCDP	0	0	3	
Simulation	IS	3	0	50	0.46
participant	RCDP	3.5	0	15	
Genuine	IS	2	0	75	0.67
participant	RCDP	2	0	25	

*IS*, immersive simulation; *RCDP*, rapid cycle deliberate practice.

* Genuine refers to experiences in actual patient care scenarios.

#### Primary Outcome: Time-based Differences

Although there were trends toward shorter mean times to completion of critical actions for RCDP vs IS groups, we observed only one category with a statistically significant difference: CPR mean pause duration in seconds was 6.20 vs 14.20 seconds (*P* = 0.01) in RCDP vs IS groups (Table 2).

**Table 2.** Rapid cycle deliberate practice vs immersive simulation time in seconds.

	RCDP mean time (±SD)	IS mean time (±SD)	*P*-value
First pulse check	4.00 (±1.00)	5.60 (±1.52)	0.25
First chest compression	12.40 (±3.13)	15.20 (±2.95)	0.27
Backboard placement	40.40 (±31.33)	193.40 (±183.36)	0.25
Pad placement	66.40 (±12.56)	74.80 (±20.75)	0.46
First rhythm analysis	73.60 (±13.50)	111.20 (±37.63)	0.25
First defibrillation	93.00 (±17.46)	150.60 (±63.49)	0.25
First epinephrine	131.60 (±28.75)	158.20 (±55.21)	0.41
Pause duration	6.20 (±2.07)	14.20 (±6.53)	0.01
Antiarryhthmic	376.60 (±94.25)	438.80 (±99.19)	0.41

*IS*, immersive simulation; *RCDP*, rapid cycle deliberate practice. Time is in seconds.

#### Secondary Outcome: Ability and Stress

For stress levels and self-reported ability, learners provided ratings on a five-point Likert-type scale. We present the mean values in Tables 3 and 4. Prior to training, RCDP and IS learners rated their anticipated stress of leading and participating in CPR similarly (4.36 vs 4.00 (*P* = 0.44); 3.18 vs 3.00 (*P* = 0.08), respectively). The RCDP and IS learners also rated their pre-training ability to lead as well as participate in the event similarly (2.50 vs 2.37 (*P* = 0.75); 3.61 vs 3.52 (*P* = 0.59) (Table 3). There was no significant difference in the anticipated stress levels of future events following training, whether considering the role of leader (*P* = 0.93) or participant (*P* = 0.98) (Table 4). Similarly, there was no significant difference in experienced stress as a leader between RCDP and IS learners (*P* = 0.93) and the overall effectiveness of the experience was rated similarly between groups (*P* = 0.09). However, RCDP learners reported lower levels of experienced stress as a participant (*P* = 0.01) (Tables 3, 4). When we compared pre- and post-training responses regarding anticipated stress, the anticipated stress of future resuscitation experiences dropped significantly for both leader and participant categories following training, regardless of instructional method.

**Table 3.** Pre-simulation mean Likert-type ratings.

	Group	Median	Min	Max	*P*-value
Ability to lead	IS	2	1	4	0.75
	RCDP	3	1	3	
Ability to participate	IS	3	3	5	0.59
	RCDP	4	2	5	
Stress anticipated as leader	IS	4	3	5	0.44
	RCDP	4	3	5	
Stress anticipated as participant	IS	4	2	4	0.08
	RCDP	3	2	5	

*IS*, immersive simulation; *RCDP*, rapid cycle deliberate practice.

**Table 4.** Post-simulation mean Likert-type ratings.

	Group	Median	Min	Max	*P*-value
Ability to lead	IS	3	3	5	0.77
	RCDP	3	3	4	
Ability to participate	IS	4	3	5	0.01
	RCDP	4.5	4	5	
Stress anticipated as leader	IS	3	2	5	0.93
	RCDP	3	2	4	
Stress anticipated as participant	IS	2	1	3	0.98
	RCDP	2	1	4	
Stress experienced as leader	IS	3	3	5	0.93
	RCDP	3	2	5	
Stress experienced as participant	IS	3	2	4	0.01
	RCDP	2	1	3	
Overall effectiveness	IS	4	3	5	0.09
	RCDP	5	4	5	

*IS*, immersive simulation; *RCDP*, rapid cycle deliberate practice.

## DISCUSSION

Learners receiving RCDP instruction showed a significantly shortened pause duration, reduced stress, and improved self-perceived CPR skills compared to IS. The RCDP instruction also shortened various time-based ACLS metrics, although statistical significance was not reached due to the small sample size. A reduced pause duration carries notable clinical significance. Pause duration is an important metric of high-quality CPR and is associated with improved patient outcomes.^23^ ^,^ ^24^ Reduced pause duration has a significant impact on terminating arrhythmias and increasing return of spontaneous circulation, while increased pause duration is associated with a decrease in survival.^26^

Although there is a paucity of literature comparing RCDP to IS in the care of an adult patient, what little data that does exist demonstrates improvements in chest compression fraction in RCDP vs IS groups.^20^ Many of these prior studies were done in pediatrics, but the results should have clinically similar interpretations as those completed in adults.^13^ ^–^ ^16^ Hunt et al conducted the only prior study examining time-based metrics as a surrogate for proficiency and found RCDP to be superior for instruction of BLS interventions in junior medical students.^27^ Our results add to this work through the further examination of time-based metrics and learner perceptions. Although limited, these results lend further credibility to the argument that RCDP may be superior to IS for ACLS training.

While RCDP-trained learners in our study exhibited trends toward other favorable ACLS metrics, there were no other statistically significant differences. Prior work has demonstrated improvement in time to defibrillation, initial chest compression, and backboard placement with RCDP training in pediatric resuscitations.^13^ ^,^ ^15^ ^,^ ^20^ Our work does not independently support these findings; however, our trends are in line with existing literature.

Time to first defibrillation suggested favorability in the RCDP group (93 vs 150 seconds [sec]), although differences did not reach statistical significance. This distinction is important, however, as the RCDP group was able perform this action within the AHA’s “Get with the Guidelines” recommendation of first defibrillation in less than two minutes. Similarly, time to first epinephrine administration in RCDP vs IS (131 vs 158 sec), suggests reduced time in the RCDP group without reaching statistical significance. Both groups performed within the five-minute metric outline from “Get with the Guidelines” recommendations. As both groups performed well with this action, obtaining statistical significance may prove difficult. It is unclear why other metrics such as pad placement or administration antiarrhythmic showed no significant change between groups. These actions are dependent on a variety of factors in a team focused on CPR, and as Lemke et al suggest, they may be difficult to measure effectively.^15^

As previously noted, our study was underpowered, which played a role in the absence of statistically significant differences for many of our outcome measures. The Likert-scale measures were better powered, as they represented 55 individual survey responses as opposed to the 10 total teams divided in two for each instructional method. For comparison, Hunt et al studied the performance of 81 individual pediatric residents who participated in the post-intervention assessment and found that RCDP improved learner confidence, but there was no control group for comparison or power calculation.^13^ De Castro et al used five teams for their RCDP group and four teams for their control group, with an 80% power to detect a 20% difference in the primary outcome. The authors found a higher chest compression fraction and shorter times to rhythm identification/defibrillation in the RCDP group. However, due to data loss they were unable to achieve the planned power.^20^ Lemke et al studied the greatest number of learners, with 102 participants in 21 teams for their control cohort, and 108 participants in 20 teams for their RCDP cohort and found that RCDP groups demonstrated shorter times to defibrillation. While no formal power calculation was performed, Lemke’s work appears to be the best powered thus far.^15^ Future work should include more robust powering with larger sample sizes, which will likely require inter-institutional collaboration.

Another factor contributing to our inability to detect significant differences in many time-based metrics may be the learner level studied. By its very nature, RCDP serves as a method to develop perfect practice. Providing the learner with real-time feedback and coaching builds micro-skill development and mastery, as opposed to proficiency alone. This study focused on PGY-1 residents for two reasons. First, in an effort to avoid confounding by variations in training, we studied PGY-1 level learners in their first month of residency. Second, we excluded advanced learners due to concerns that their involvement would confound the study of the junior learner through advancing the performance of the entire group. Conversely, prior work that found differences in similar categories evaluated learners from PGY levels 1–3 or studied larger learner groups.^14^ ^,^ ^15^ ^,^ ^27^ Therefore, true skill mastery may be more attainable through the inclusion of more advanced learners and may contribute to more statistically significant results.^13^ ^,^ ^15^ Conversely, the inclusion of more advanced learners may influence the entire group, leading to a more uniform performance. This may limit or reduce observable differences between instructional methods.

Hunt et al also notes a dose response with RCDP (ie, increasing experience and repetition fosters improved performance and skill mastery).^13^ We studied the learners’ first performance, but we did not conduct additional simulated experiences beyond this. Further repetition may have expanded differences in RCDP and IS groups.

A common goal of simulation in medical education is to reduce the stress and anxiety experienced by the learner, and this is especially true for high-stakes scenarios such as the care of a pulseless patient. However, the simulation experience can be independently stressful for learners, and prior work has suggested that RCDP-based instruction may provide an overall preferred experience. This is well illustrated by the work of Chancey et al, whose learners expressed a preference for the frequent interruptions and improved sense of emotional security associated with RCDP instruction.^25^ Chancey’s learners also reported increased confidence in their own resuscitation skills. Our results support these findings, demonstrating an increased confidence in ability to participate in the RCDP groups. Similarly, our learners reported lower stress levels experienced during their RCDP-based simulation.

## LIMITATIONS

Due to the study’s nature, blinding participants and facilitators was not possible. Skill retention was not assessed, and the small sample size limits generalizability. Additionally, while all facilitators had undergone standardized training in both instructional methods, individual facilitators may have been more effective at one strategy vs the other. All participating residents completed a standard ACLS course in the two weeks preceding the study. Also, most of the residents had significant experience as part of resuscitation teams (Table 1). As a result, there may have been less of a difference in performance between the two groups. Our study found RCDP was well received by our learners, but the data is limited by learner evaluation at Kirkpatrick levels I and II. While we believe learner perceptions in instruction are important for engagement, future investigations should focus on objective impacts and clinical performance with patient-oriented outcomes.

Surveys were not based on any prior survey instrument but were created, reviewed, and edited by the research team. The surveys were novel instruments, and we did not obtain validity evidence prior to their use. Recall bias was minimized through the implementation of surveys immediately following instruction and performance of the learners. We were unable to eliminate the effects of social desirability bias for our learners and suspect that learners would tend to report improved performance regardless of instructional method. However, the potential for this bias existed in both RCDP and IS groups. Sampling and non-response bias were not factors secondary to our 100% response rate, but due to the nature of our five-point Likert-type question scale, the potential for neutral bias exists.

Due to the frequent interruptions associated with the RCDP method, RCDP participants were able to rotate through each role on the resuscitation team. However, IS groups did not have an opportunity to change roles as a part of their training, and this introduces a confounder in comparing the learner experience as well as proficiency between these instructional methods.

Finally, this study focused on time to completion of critical actions but did not assess the quality of those actions, including factors such as chest compression fraction (CCF). However, CCF has been previously studied and found to be superior in groups undergoing RCDP-based instruction as compared to standard IS.^13^ ^,^ ^20^ ^,^ ^27^

## CONCLUSION

Rapid cycle deliberate practice was favored by learners for ACLS-based CPR instruction, improving self-perceived skills and reducing pause duration. This suggests RCDP is a valid strategy to teach residents ACLS-based CPR and supports further investigation of RCDP in other settings.
